# Supplementary material for: The in vitro and in vivo effects of constitutive light expression on a bioluminescent strain of the mouse enteropathogen Citrobacter rodentium
Source: PeerJ. 2016 Jun 22;4:e2130. doi: 10.7717/peerj.2130 (PMC4924136; doi:10.7717/peerj.2130)
Supplement: Table S1 — Substrates and positions within the 96 well plates of the Biolog PM plates used in this study. [file peerj-04-2130-s002.docx]

**Supplementary Table 1. BIOLOG Phenotypic Microarray assays.**

Substrates and positions within the 96 well plates of the Biolog PM plates used in this study

| PM1 – Carbon Sources | A1, Negative Control; A2, L-Arabinose; A3, N-Acetyl-D Glucosamine; A4, D-Saccharic Acid; A5, Succinic Acid; A6, D-Galactose; A7, L-Aspartic Acid; A8, L-Proline; A9, D-Alanine; A10, D-Trehalose; A11, D-Mannose; A12, Dulcitol; B1, D-Serine; B2, D-Sorbitol; B3, Glycerol; B4, L-Fucose; B5, D-Glucuronic Acid; B6, D-Gluconic Acid; B7, D,L-α-Glycerol Phosphate; B8, D-Xylose; B9, L-Lactic Acid; B10, Formic Acid; B11, D-Mannitol; B12, L-Glutamic Acid; C1, D-Glucose-6-Phosphate; C2, D-Galactonic Acid-γ-Lactone; C3, D,L-Malic Acid; C4, D-Ribose; C5, Tween 20; C6, L-Rhamnose; C7, D-Fructose; C8, Acetic Acid; C9, α-D-Glucose; C10, Maltose; C11, D-Melibiose; C12, Thymidine; D1, L-Asparagine; D2, D-Aspartic Acid; D3, D-Glucosaminic Acid; D4, 1,2- Propanediol; D5, Tween 40; D6, α -Keto-Glutaric Acid; D7, α -Keto-Butyric Acid; D8, α -Methyl-D Galactoside; D9, α -D-Lactose; D10, Lactulose; D11, Sucrose; D12, Uridine; E1, L-Glutamine; E2, m-Tartaric Acid; E3, D-Glucose-1-Phosphate; E4, D-Fructose-6-Phosphate; E5, Tween 80; E6, α-Hydroxy Glutaric Acid-α-Lactone; E7, α-Hydroxy Butyric Acid; E8, α-Methyl-DGlucoside; E9, Adonitol; E10, Maltotriose; E11, 2-Deoxy Adenosine; E12, Adenosine; F1, Glycyl-L-Aspartic Acid; F2, Citric Acid; F3, m-Inositol; F4, D-Threonine; F5, Fumaric Acid; F6, Bromo Succinic Acid; F7, Propionic Acid; F8, Mucic Acid; F9, Glycolic Acid; F10, Glyoxylic Acid; F11, D-Cellobiose; F12, Inosine; G1, Glycyl-L-Glutamic Acid; G2, Tricarballylic Acid; G3, L-Serine; G4, L-Threonine; G5,L-Alanine; G6, L-Alanyl-Glycine; G7, Acetoacetic Acid; G8, N-Acetyl-β-D-Mannosamine; G9, Mono Methyl Succinate; G10, Methyl Pyruvate; G11, D-Malic Acid; G12, L-Malic Acid; H1, Glycyl-L-Proline; H2, p-Hydroxy Phenyl Acetic Acid; H3, m-Hydroxy Phenyl Acetic Acid; H4, Tyramine; H5, D-Psicose; H6, L-Lyxose; H7, Glucuronamide; H8, Pyruvic Acid; H9, L-Galactonic Acid-γ-Lactone; H10, D-Galacturonic Acid; H11, Phenylethylamine; H12, 2-Amino Ethanol. |
| --- | --- |
| PM2 – Carbon Sources | A1, Negative Control; A2, Chondroitin Sulfate C; A3, α-Cyclodextrin; A4, β-Cyclodextrin; A5, γ-Cyclodextrin; A6, Dextrin; A7, Gelatin; A8, Glycogen; A9, Inulin; A10, Laminarin; A11, Mannan; A12, Pectin; B1, N-Acetyl-D Galactosamine; B2, N-Acetyl Neuraminic Acid; B3, β-D-Allose; B4, Amygdalin; B5, D-Arabinose; B6, D-Arabitol; B7, L-Arabitol; B8, Arbutin; B9, 2-Deoxy-D Ribose; B10, i-Erythritol; B11, D-Fucose; B12,3-0-β-D-Galactopyranosyl-D Arabinose; C1, Gentiobiose; C2, L-Glucose; C3, Lactitol; C4, D-Melezitose; C5, Maltitol; C6, a-Methyl-D Glucoside; C7, β-Methyl-D Galactoside; C8, 3-Methyl Glucose; C9, β -Methyl-D Glucuronic Acid; C10, α-Methyl-D Mannoside; C11, β -Methyl-D Xyloside; C12, Palatinose; D1, D-Raffinose; D2, Salicin; D3, Sedoheptulosan; D4, L-Sorbose; D5, Stachyose; D6, D-Tagatose; D7, Turanose; D8, Xylitol; D9, N-Acetyl-D Glucosaminitol; D10, γ-Amino Butyric Acid; D11, δ-Amino Valeric Acid; D12, Butyric Acid; E1, Capric Acid; E2, Caproic Acid; E3, Citraconic Acid; E4, Citramalic Acid; E5, D-Glucosamine; E6, 2-Hydroxy Benzoic Acid; E7, 4-Hydroxy Benzoic Acid; E8, β-Hydroxy Butyric Acid; E9, δ-Hydroxy Butyric Acid; E10, a-Keto-Valeric Acid; E11, Itaconic Acid; E12, 5-Keto-D Gluconic Acid; F1, D-Lactic Acid Methyl Ester; F2, Malonic Acid; F3, Melibionic Acid; F4, Oxalic Acid; F5, Oxalomalic Acid; F6, Quinic Acid; F7, D-Ribono-1,4-Lactone; F8, Sebacic Acid; F9, Sorbic Acid; F10, Succinamic Acid; F11, D-Tartaric Acid; F12, L-Tartaric Acid; G1, Acetamide; G2, L-Alaninamide; G3, N-Acetyl-L Glutamic Acid; G4, L-Arginine; G5, Glycine; G6, L-Histidine; G7, L-Homoserine; G8, Hydroxy-L Proline; G9, L-Isoleucine; G10, L-Leucine; G11, L-Lysine; G12, L-Methionine; H1, L-Ornithine; H2, L-Phenylalanine; H3, L-Pyroglutamic Acid; H4, L-Valine; H5, D,L-Carnitine; H6, Sec-Butylamine; H7, D.L-Octopamine; H8, Putrescine; H9, Dihydroxy Acetone; H10, 2,3-Butanediol; H11, 2,3-Butanone; H12, 3-Hydroxy 2-Butanone. |
| PM3 – Nitrogen Sources | A1, Negative Control; A2, Ammonia; A3, Nitrite; A4, Nitrate; A5, Urea; A6, Biuret; A7, L-Alanine; A8, L-Arginine; A9, L-Asparagine; A10, L-Aspartic Acid; A11, L-Cysteine; A12, L-Glutamic Acid; B1, L-Glutamine; B2, Glycine; B3, L-Histidine; B4, L-Isoleucine; B5, L-Leucine; B6, L-Lysine; B7, L-Methionine; B8, L-Phenylalanine; B9, L-Proline; B10, L-Serine; B11, L-Threonine; B12, L-Tryptophan; C1, L-Tyrosine; C2, L-Valine; C3, D-Alanine; C4, D-Asparagine; C5, D-Aspartic Acid; C6, D-Glutamic Acid; C7, D-Lysine; C8, D-Serine; C9, D-Valine; C10, L-Citrulline; C11, L-Homoserine; C12, L-Ornithine; D1, N-Acetyl-L Glutamic Acid; D2, N-Phthaloyl-L Glutamic Acid; D3, L-Pyroglutamic Acid; D4, Hydroxylamine; D5, Methylamine; D6, N-Amylamine; D7, N-Butylamine; D8, Ethylamine; D9, Ethanolamine; D10, Ethylenediamine; D11, Putrescine; D12, Agmatine; E1, Histamine; E2, β-Phenylethylamine; E3, Tyramine; E4, Acetamide; E5, Formamide; E6, Glucuronamide; E7, D,L-Lactamide; E8, D-Glucosamine; E9, D-Galactosamine; E10, D-Mannosamine; E11, N-Acetyl-D Glucosamine; E12, N-Acetyl-D Galactosamine; F1, N-Acetyl-D Mannosamine; F2, Adenine; F3, Adenosine; F4, Cytidine; F5, Cytosine; F6, Guanine; F7, Guanosine; F8, Thymine; F9, Thymidine; F10, Uracil; F11, Uridine; F12, Inosine; G1, Xanthine; G2, Xanthosine; G3, Uric Acid; G4, Alloxan; G5, Allantoin; G6, Parabanic Acid; G7, D,L-α-Amino-N Butyric Acid; G8, β-Amino-N Butyric Acid; G9, ε-Amino-N Caproic Acid; G10, D,L-α-Amino Caprylic Acid; G11, δ-Amino-N Valeric Acid; G12, α-Amino-N Valeric Acid; H1, Ala-Asp; H2, Ala-Gln; H3, Ala-Glu; H4, Ala-Gly; H5, Ala-His; H6, Ala-Leu; H7, Ala-Thr; H8, Gly-Asn; H9, Gly-Gln; H10, Gly-Glu; H11, Gly-Met; H12, Met-Ala. |
| PM4 – Phosphorus and Sulfur Sources | A1, Negative Control; A2, Phosphate; A3, Pyrophosphate; A4, Trimetaphosphate; A5, Tripolyphosphate; A6, Triethyl Phosphate; A7, Hypophosphite; A8, Adenosine-2’-monophosphate; A9, Adenosine-3’-monophosphate; A10, Adenosine-5’-monophosphate; A11, Adenosine-2’,3’-cyclic monophosphate; A12, Adenosine-3’,5’-cyclic monophosphate; B1, Thiophosphate; B2, Dithiophosphate; B3, D,L-α-Glycerol Phosphate; B4, β-Glycerol Phosphate; B5, Carbamyl Phosphate; B6, D-2-Phospho Glyceric Acid; B7, D-3-Phospho Glyceric Acid; B8, Guanosine-2’-monophosphate; B9, Guanosine-3’-monophosphate; B10, Guanosine-5’-monophosphate; B11, Guanosine-2’,3’-cyclic monophosphate; B12, Guanosine-3’,5’-cyclic monophosphate; C1, Phosphoenol Pyruvate; C2, Phospho Glycolic Acid; C3, D-Glucose-1-Phosphate; C4, D-Glucose-6-Phosphate; C5, 2-Deoxy-D Glucose 6-Phosphate; C6, D-Glucosamine-6-Phosphate; C7, 6-Phospho Gluconic Acid; C8, Cytidine-2’-monophosphate; C9, Cytidine-3’-monophosphate; C10, Cytidine-5’-monophosphate; C11, Cytidine-2’,3’-cyclic monophosphate; C12, Cytidine-3’,5’-cyclic monophosphate; D1, D-Mannose-1-Phosphate; D2, D-Mannose-6-Phosphate; D3, Cysteamine-S Phosphate; D4, Phospho-L Arginine; D5, O-Phospho-D Serine; D6, O-Phospho-L Serine; D7, O-Phospho-L Threonine; D8, Uridine-2’-monophosphate; D9, Uridine-3’-monophosphate; D10, Uridine-5’-monophosphate; D11, Uridine-2’,3’-cyclic monophosphate; D12, Uridine-3’,5’-cyclic monophosphate; E1, O-Phospho-D Tyrosine; E2, O-Phospho-L Tyrosine; E3, Phosphocreatine; E4, Phosphoryl Choline; E5, O-Phosphoryl Ethanolamine; E6, Phosphono Acetic Acid; E7, 2-Aminoethyl Phosphonic Acid; E8, Methylene Diphosphonic Acid; E9, Thymidine-3’-monophosphate; E10, Thymidine-5’-monophosphate; E11, Inositol Hexaphosphate; E12, Thymidine 3’,5’-cyclic monophosphate; F1, Negative Control; F2, Sulfate; F3, Thiosulfate; F4, Tetrathionate; F5, Thiophosphate; F6, Dithiophosphate; F7, L-Cysteine; F8, D-Cysteine; F9, L-Cysteinyl Glycine; F10, L-Cysteic Acid; F11, Cysteamine; F12, L-Cysteine Sulfinic Acid; G1, N-Acetyl-L Cysteine; G2, S-Methyl-L Cysteine; G3, Cystathionine; G4, Lanthionine; G5, Glutathione; G6, D,L-Ethionine; G7, L-Methionine; G8, D-Methionine; G9, Glycyl-L Methionine; G10, N-Acetyl-D,L Methionine; G11, L- Methionine Sulfoxide; G12, L-Methionine Sulfone; H1, L-Djenkolic Acid; H2, Thiourea; H3, 1-Thio-β-D Glucose; H4, D,L-Lipoamide; H5, Taurocholic Acid; H6, Taurine; H7, Hypotaurine; H8, p-Amino Benzene Sulfonic Acid; H9, Butane Sulfonic Acid; H10, 2-Hydroxyethane Sulfonic Acid; H11, Methane Sulfonic Acid; H12, Tetramethylene Sulfone. |
| PM5 – Nutrient Supplements | A1, Negative Control; A2, Positive Control; A3, L-Alanine; A4, L-Arginine; A5, L-Asparagine; A6, L-Aspartic Acid; A7, L-Cysteine, A8, L-Glutamic Acid; A9, Adenosine-3’,5’-cyclic monophosphate; A10, Adenine; A11, Adenosine; A12, 2’-Deoxy Adenosine; B1, L-Glutamine; B2, Glycine; B3, L-Histidine; B4, L-Isoleucine; B5, L-Leucine; B6, L-Lysine; B7, L-Methionine; B8, L-Phenylalanine; B9, Guanosine-3’,5’-cyclic monophosphate; B10, Guanine; B11, Guanosine; B12, 2’-Deoxy Guanosine; C1, L-Proline; C2, L-Serine; C3, L-Threonine; C4, L-Tryptophan; C5, L-Tyrosine; C6, L-Valine; C7, L-isoleucine + L-Valine; C8, trans-4-Hydroxy L-Proline; C9, (5) 4-AminoImidazole-4(5)-Carboxamide; C10, Hypoxanthine; C11, Inosine; C12, 2’-Deoxy Inosine; D1, L-Ornithine; D2, L-Citrulline; D3, Chorismic Acid; D4, (-)Shikimic Acid; D5, L-Homoserine Lactone; D6, D-Alanine; D7, D-Aspartic Acid; D8, D-Glutamic Acid; D9, D,L-α,ε-Diaminopimelic Acid; D10, Cytosine; D11, Cytidine; D12, 2’-Deoxy Cytidine; E1, Putrescine; E2, Spermidine; E3, Spermine; E4, Pyridoxine; E5, Pyridoxal; E6, Pyridoxamine; E7, β-Alanine; E8, D-Pantothenic Acid; E9, Orotic Acid; E10, Uracil; E11, Uridine; E12, 2’-Deoxy Uridine; F1, Quinolinic Acid; F2, Nicotinic Acid; F3, Nicotinamide; F4, β-Nicotinamide Adenine Dinucleotide; F5, δ-Amino Levulinic Acid; F6, Hematin  F7, Deferoxamine; Mesylate; F8, D-(+)-Glucose; F9, N-Acetyl D-Glucosamine; F10, Thymine; F11, Glutathione (reduced form); F12, Thymidine; G1, Oxaloacetic Acid; G2, D-Biotin; G3, CyanoCobalamine; G4, p-Amino Benzoic Acid; G5, Folic Acid; G6, Inosine +Thiamine; G7, Thiamine; G8, Thiamine Pyrophosphate; G9, Riboflavin; G10, Pyrrolo-Quinoline Quinone; G11, Menadione; G12, m-Inositol; H1, Butyric Acid; H2, D,L-α-Hydroxy Butyric Acid; H3, α-Keto Butyric Acid; H4, Caprylic Acid; H5, D,L- α-Lipoic Acid (oxidized form); H6, D,L-Mevalonic Acid; H7, D,L-Carnitine; H8, Choline; H9, Tween 20; H10, Tween 40; H11, Tween 60; H12, Tween 80. |
| PM6 – Peptide Nitrogen sources | A1, Negative Control; A2, Positive Control: L-Glutamine; A3, Ala-Ala; A4, Ala-Arg; A5, Ala-Asn; A6, Ala-Glu; A7, Ala-Gly; A8, Ala-His; A9, Ala-Leu; A10, Ala-Lys; A11, Ala-Phe; A12, Ala-Pro; B1, Ala-Ser; B2, Ala-Thr; B3, Ala-Trp; B4, Ala-Tyr; B5, Arg-Ala; B6, Arg-Arg; B7, Arg-Asp; B8, Arg-Gln; B9, ; rg-Glu; B10, Arg-Ile; B11, Arg-Leu; B12, Arg-Lys; C1, Arg-Met; C2, Arg-Phe; C3, Arg-Ser; C4, Arg-; rp; C5, Arg-Tyr; C6, Arg-Val; C7, Asn-Glu; C8, Asn-Val; C9, Asp-Asp; C10, Asp-Glu; C11, Asp-Leu; C12, Asp-Lys; D1, Asp-Phe; D2, Asp-Trp; D3, Asp-Val; D4, Cys-Gly; D5, Gln-Gln ;D6, Gln-Gly; D7, Glu-Asp; D8, Glu-Glu; D9, Glu-Gly; D10, Glu-Ser; D11, Glu-Trp; D12, Glu-Tyr; E1, Glu-Val; E2, Gly-Ala; E3, Gly-Arg; E4, Gly-Cys; E5, Gly-Gly; E6, Gly-His; E7, Gly-Leu; E8, Gly-Lys; E9, Gly-Met; E10, Gly-Phe; E11, Gly-Pro; E12, Gly-Ser; F1, Gly-Thr; F2, Gly-Trp; F3, Gly-Tyr; F4, Gly-Val; F5, His-Asp; F6, His-Gly; F7, His-Leu; F8, His-Lys; F9, His-Met; F10, His-Pro; F11 ,His-Ser; F12, His-Trp; G1, His-Tyr; G2, His-Val; G3, Ile-Ala; G4, Ile-Arg; G5, Ile-Gln; G6, Ile-Gly; G7, Ile-His; G8, Ile-Ile; G9, Ile-Met; G10, Ile-Phe; G11, Ile-Pro; G12, Ile-Ser; H1, lle-Trp; H2, Ile-Tyr; H3, Ile-Val; H4, Leu-Ala; H5, Leu-Arg; H6, Leu-Asp; H7, Leu-Glu; H8, Leu-Gly; H9, Leu-Ile; H10, Leu-Leu; H11, Leu-Met; H12, Leu-Phe. |
| PM7 – Peptide Nitrogen sources | A1, Negative Control; A2, Positive Control: L-Glutamine; A3, Leu-Ser; A4, Leu-Trp; A5, Leu-Val; A6, Lys-Ala; A7, Lys-Arg; A8, Lys-Glu; A9, Lys-Ile; A10, Lys-Leu; A11, Lys-Lys; A12, Lys-Phe; B1, Lys-Pro; B2, Lys-Ser; B3, Lys-Thr; B4, Lys-Trp; B5 ,Lys-Tyr; B6, Lys-Val; B7, Met-Arg; B8, Met-Asp; B9, Met-Gln; B10, Met-Glu; B11, Met-Gly; B12, Met-His; C1, Met-Ile; C2, Met-Leu; C3, Met-Lys; C4, Met-Met; C5, Met-Phe; C6, Met-Pro; C7, Met-Trp; C8, Met-Val; C9, Phe-Ala; C10, Phe-Gly; C11, Phe-Ile; C12, Phe-Phe; D1, Phe-Pro; D2, Phe-Ser; D3, Phe-Trp; D4, Pro-Ala; D5, Pro-Asp; D6, Pro-Gln; D7, Pro-Gly; D8, Pro-Hyp; D9, Pro-Leu; D10, Pro-Phe; D11, Pro-Pro; D12, Pro-Tyr; E1, Ser-Ala; E2, Ser-Gly; E3, Ser-His; E4, Ser-Leu; E5, Ser-Met; E6, Ser-Phe; E7, Ser-Pro; E8, Ser-Ser; E9, Ser-Tyr; E10, Ser-Val; E11, Thr-Ala; E12, Thr-Arg; F1, Thr-Glu; F2, Thr-Gly; F3, Thr-Leu; F4, Thr-Met; F5, Thr-Pro; F6, Trp-Ala; F7, Trp-Arg; F8, Trp-Asp; F9, Trp-Glu; F10, Trp-Gly; F11, Trp-Leu; F12, Trp-Lys; G1, Trp-Phe; G2, Trp-Ser; G3, Trp-Trp; G4, Trp-Tyr; G5, Tyr-Ala; G6, Tyr-Gln; G7, Tyr-Glu; G8, Tyr-Gly G9, Tyr-His; G10, Tyr-Leu; G11, Tyr-Lys; G12, Tyr-Phe; H1, Tyr-Trp; H2, Tyr-Tyr; H3, Val-Arg; H4, Val-Asn; H5, Val-Asp; H6, Val-Gly; H7, Val-His; H8, Val-Ile; H9, Val-Leu; H10, Val-Tyr; H11, Val-Val; H12, ϒ-Glu-Gly. |
| PM8 – Peptide Nitrogen sources | A1, Negative Control; A2, Positive Control: L-Glutamine; A3, Ala-Asp; A4, Ala-Gln; A5, Ala-lle; A6, Ala-Met; A7, Ala-Val; A8, Asp-Ala; A9, Asp-Gln; A10, Asp-Gly; A11, Glu-Ala; A12, Gly-Asn; B1, Gly-Asp; B2, Gly-lle; B3, His-Ala; B4, His-Glu; B5, His-His; B6, Ile-Asn; B7, Ile-Leu; B8, Leu-Asn; B9, Leu-His; B10, Leu-Pro; B11, Leu-Tyr; B12, Lys-Asp; C1, Lys-Gly; C2, Lys-Met; C3, Met-Thr; C4, Met-Tyr; C5, Phe-Asp; C6, Phe-Glu; C7, Gln-Glu; C8, Phe-Met; C9, Phe-Tyr; C10, Phe-Val; C11, Pro-Arg; C12, Pro-Asn; D1, Pro-Glu; D2, Pro-lle; D3, Pro-Lys; D4, Pro-Ser; D5, Pro-Trp; D6, Pro-Val; D7, Ser-Asn; D8, Ser-Asp; D9, Ser-Gln; D10, Ser-Glu; D11, Thr-Asp; D12, Thr-Gln; E1, Thr-Phe; E2, Thr-Ser; E3, Trp-Val; E4, Tyr-lle; E5, Tyr-Val; E6, Val-Ala; E7, Val-Gln; E8, Val-Glu; E9, Val-Lys; E10, Val-Met; E11, Val-Phe; E12, Val-Pro; F1, Val-Ser; F2, β-Ala-Ala; F3, β-Ala-Gly; F4, β-Ala-His; F5, Met- β-Ala; F6, β-Ala-Phe; F7, D-Ala-D-Ala; F8, D-Ala-Gly; F9, D-Ala-Leu; F10, D-Leu-D-Leu; F11, D-Leu-Gly; F12, D-Leu-Tyr; G1, ϒ-Glu-Gly; G2, ϒ-D-Glu-Gly; G3, Gly-D-Ala; G4, Gly-D-Asp; G5, Gly-D-Ser; G6, Gly-D-Thr; G7, Gly-D-Val; G8, Leu-β-Ala; G9, Leu-D-Leu; G10, Phe-β-Ala; G11, Ala-Ala-Ala; G12, D-Ala-Gly-Gly; H1, Gly-Gly-Ala; H2, Gly-Gly-D-Leu; H3, Gly-Gly-Gly; H4, Gly-Gly-lle; H5, Gly-Gly-Leu; H6, Gly-Gly-Phe; H7, Val-Tyr-Val; H8, Gly-Phe-Phe; H9, Leu-Gly-Gly; H10, Leu-Leu-Leu; H11, Phe-Gly-Gly; H12, Tyr-Gly-Gly. |
| PM9 – Osmolytes | A1, NaCl 1%; A2, NaCl 2%; A3, NaCl 3%; A4, NaCl 4%; A5, NaCl 5%; A6, NaCl 5.5%; A7, NaCl 6%; A8, NaCl 6.5%; A9, NaCl 7%; A10, NaCl 8%; A11, NaCl 9%; A12, NaCl 10%; B1, NaCl 6%; B2, NaCl 6% +Betaine; B3, NaCl 6% +N-N Dimethyl Glycine; B4, NaCl 6% + Sarcosine; B5, NaCl 6% + Dimethyl sulphonyl propionate; B6, NaCl 6% + MOPS; B7, NaCl 6% + Ectoine; B8, NaCl 6% + Choline; B9, NaCl 6% + Phosphoryl Choline; B10, NaCl 6% + Creatine; B11, NaCl 6% + Creatinine; B12, NaCl 6% + L- Carnitine; C1, NaCl 6% + KCl; C2, NaCl 6% + L-Proline; C3, NaCl 6% + N-Acetyl L-Glutamine; C4, NaC1 6% + β-Glutamic Acid; C5 ,NaC1 6% + γ–Amino –N Butyric Acid; C6, NaC1 6% + Glutathione; C7, NaCl 6% + Glycerol; C8, NaC1 6% +Trehalose; C9, NaC1 6% + TrimethylamineN-oxide; C10, NaC1 6% + Trimethylamine; C11, NaCl 6% + Octopine; C12, NaC1 6% + Trigonelline; D1, Potassium chloride 3%; D2, Potassium chloride 4%; D3, Potassium chloride 5%; D4, Potassium chloride 6%; D5, Sodium sulphate 2%; D6, Sodium sulphate 3%; D7, Sodium sulphate 4%; D8, Sodium sulphate 5%; D9, Ethylene glycol 5%; D10, Ethylene glycol 10%; D11, Ethylene glycol 15%; D12, Ethylene glycol 20%; E1, Sodium formate 1%; E2, Sodium formate 2%; E3, Sodium formate 3%; E4, Sodium formate 4%; E5, Sodium formate 5%; E6, Sodium formate 6%; E7, Urea 2%; E8, Urea 3%; E9, Urea 4%; E10, Urea 5%; E11, Urea 6%; E12, Urea 7%; F1, Sodium Lactate 1%; F2, Sodium Lactate 2%; F3, Sodium Lactate 3%; F4, Sodium Lactate 4%; F5, Sodium Lactate 5%; F6, Sodium Lactate 6%; F7, Sodium Lactate 7%; F8, Sodium Lactate 8%; F9, Sodium Lactate 9%; F10, Sodium Lactate 10%; F11, Sodium Lactate 11%; F12, Sodium Lactate 12%; G1, Sodium Phosphate pH 7 20mM; G2, Sodium Phosphate pH 7 50mM; G3, Sodium Phosphate pH 7 100mM; G4, Sodium Phosphate pH 7 200mM; G5, Sodium Benzoate pH 5.2 20mM; G6, Sodium Benzoate pH 5.2 50mM; G7, Sodium Benzoate pH5.2 100mM; G8, Sodium Benzoate pH 5.2 200mM; G9, Ammonium sulfate pH8 10mM; G10, Ammonium sulfate pH 8 20mM; G11, Ammonium sulfate pH 8 50mM; G12, Ammonium sulfate pH8 100mM; H1, Sodium Nitrate 10mM; H2, Sodium Nitrate 20mM; H3, Sodium Nitrate 40mM; H4, Sodium Nitrate 60mM; H5, Sodium Nitrate 80mM; H6, Sodium Nitrate 100mM; H7, Sodium Nitrite 10mM; H8, Sodium Nitrite 20mM; H9, Sodium Nitrite 40mM; H10, Sodium Nitrite 60mM; H11, Sodium Nitrite 80mM; H12, Sodium Nitrite 100mM. |
| PM10 – pH | A1, pH 3.5; A2, pH 4; A3, pH 4.5; A4, pH 5; A5, pH 5.5; A6, pH 6; A7, pH 7; A8, pH 8; A9, pH 8.5; A10, pH 9; A11, pH 9.5; A12, pH 10; B1, pH 4.5; B2, pH 4.5 + L-Alanine; B3, pH 4.5 + L-Arginine; B4, pH 4.5 + L-Asparagine; B5, pH 4.5 + L-Aspartic Acid; B6, pH 4.5 + L-Glutamic Acid; B7, pH 4.5 + L-Glutamine; B8, pH 4.5 + Glycine; B9, pH 4.5 + L-Histidine; B10, pH 4.5 + L-Isoleucine; B11, pH 4.5 + L-Leucine; B12, pH 4.5 + L-Lysine; C1, pH 4.5 + L-Methionine; C2, pH 4.5 + L-Phenylalanine; C3, pH 4.5 + L-Proline; C4, pH 4.5 + L-Serine; C5, pH 4.5 + L-Threonine; C6, pH 4.5 + L-Tryptophan; C7, pH 4.5 + L-Citrulline; C8, pH 4.5 + L-Valine; C9, pH 4.5 + HydroxyL-Proline; C10, pH 4.5 + L-Ornithine; C11, pH 4.5 + L-Homoarginine; C12, pH 4.5 + L-Homoserine; D-1, pH 4.5 + Anthranilic Acid; D2, pH 4.5 + L-Norleucine; D3, pH 4.5 + L-Norvaline; D4, pH 4.5 + α- Amino-N Butyric Acid; D5, pH 4.5 + p-Amino Benzoic Acid; D6, pH 4.5 + L-Cysteic Acid; D7, pH 4.5 + D-Lysine; D8, pH 4.5 + 5-Hydroxy Lysine; D9, pH 4.5 + 5-Hydroxy Tryptophan; D10, pH 4.5 + D,L-Diamino pimelic Acid; D11, pH 4.5 + Trimethylamine N-oxide; D12, pH 4.5 + Urea; E1, pH 9.5; E2, pH 9.5 + L-Alanine; E3, pH 9.5 + L-Arginine; E4, pH 9.5 + L-Asparagine; E5, pH 9.5 + L-Aspartic Acid; E6, pH 9.5 + L-Glutamic Acid; E7, pH 9.5 + L-Glutamine; E8, pH 9.5 + Glycine; E9, pH 9.5 + L-Histidine; E10, pH 9.5 + L-Isoleucine; E11, pH 9.5 + L-Leucine; E12, pH 9.5 + L-Lysine; F1, pH 9.5 + L-Methionine; F2, pH 9.5 + L-Phenylalanine; F3, pH 9.5 + L-Proline; F4, pH 9.5 + L-Serine; F5, pH 9.5 + L-Threonine; F6, pH 9.5 + L-Tryptophan; F7, pH 9.5 + L-Tyrosine; F8, pH 9.5 + L-Valine; F9, pH 9.5 + Hydroxy L-Proline; F10, pH 9.5 + L-Ornithine; F11, pH 9.5 + L-Homoarginine; F12, pH 9.5 + L-Homoserine; G1, pH 9.5 + Anthranilic acid; G2, pH 9.5 + L-Norleucine; G3, pH 9.5 + L-Norvaline; G4, pH 9.5 + Agmatine; G5, pH 9.5 + Cadaverine; G6, pH 9.5 + Putrescine; G7, pH 9.5 + Histamine; G8, pH 9.5 + Phenylethylamine; G9, pH 9.5 + Tyramine; G10, pH 9.5 + Creatine; G11, pH 9.5 + Trimethylamine N-oxide; G12, pH 9.5 + Urea; H1, X-Caprylate; H2, X–α-DGlucoside; H3, X-β-DGlucoside; H4, X-α-DGalactoside; H5, X-β-DGalactoside; H6, X-α- DGlucuronide; H7, X-β- DGlucuronide; H8, X-β-DGlucosaminide; H9, X-β-DGalactosaminide; H10, X-α-DMannoside; H11, X-PO4; H12, X-SO4. |
| PM11C – chemical | A1, Amikacin (1); A2, Amikacin (2); A3, Amikacin (3); A4, Amikacin (4); A5, Chlortetracycline (1); A6, Chlortetracycline (2) ;A7, Chlortetracycline (3); A8, Chlortetracycline (4); A9, Lincomycin (1); A10, Lincomycin (2); A11, Lincomycin (3); A12, Lincomycin (4); B1, Amoxicillin (1); B2, Amoxicillin (2); B3, Amoxicillin (3); B4, Amoxicillin (4); B5, Cloxacillin (1); B6, Cloxacillin (2); B7, Cloxacillin (3); B8, Cloxacillin (4); B9, Lomefloxacin (1); B10, Lomefloxacin (2); B11, Lomefloxacin (3); B12, Lomefloxacin (4); C1, Bleomycin (1); C2, Bleomycin (2); C3, Bleomycin (3); C4, Bleomycin (4); C5, Colistin (1); C6, Colistin (2); C7, Colistin (3); C8, Colistin (4); C9, Minocycline (1); C10, Minocycline (2); C11, Minocycline (3); C12, Minocycline (4); D1, Capreomycin (1); D2, Capreomycin (2); D3, Capreomycin (3); D4, Capreomycin (4); D5, Demeclocycline (1); D6, Demeclocycline (2); D7, Demeclocycline (3); D8, Demeclocycline (4); D9, Nafcillin (1); D10, Nafcillin (2); D11, Nafcillin (3); D12, Nafcillin (4); E1, Cefazolin (1); E2, Cefazolin (2); E3, Cefazolin (3); E4, Cefazolin (4); E5, Enoxacin (1); E6, Enoxacin (2); E7, Enoxacin (3); E8, Enoxacin (4); E9, Nalidixic acid (1); E10, Nalidixic acid (2); E11, Nalidixic acid (3); E12, Nalidixic acid (4); F1, Chloramphenicol (1); F2, Chloramphenicol (2); F3, Chloramphenicol (3); F4, Chloramphenicol (4); F5, Erythromycin (1); F6, Erythromycin (2); F7, Erythromycin (3); F8, Erythromycin (4); F9, Neomycin (1); F10, Neomycin (2); F11, Neomycin (3); F12, Neomycin (4); G1, Ceftriaxone (1); G2, Ceftriaxone (2); G3, Ceftriaxone (3); G4, Ceftriaxone (4); G5, Gentamicin (1); G6, Gentamicin (2); G7, Gentamicin (3); G8, Gentamicin (4); G9, Potassium tellurite (1); G10, Potassium tellurite (2); G11, Potassium tellurite (3); G12, Potassium tellurite (4); H1, Cephalothin (1); H2, Cephalothin (2); H3, Cephalothin (3); H4, Cephalothin (4); H5, Kanamycin (1); H6, Kanamycin (2); H7, Kanamycin (3); H8, Kanamycin (4); H9, Ofloxacin (1); H10, Ofloxacin (2); H11, Ofloxacin (3); H12, Ofloxacin (4). |
| PM12B – chemical | A1, Penicillin G (1); A2, Penicillin G (2); A3, Penicillin G (3); A4, Penicillin G (4); A5, Tetracycline (1); A6, Tetracycline (2); A7, Tetracycline (3); A8, Tetracycline (4); A9, Carbenicillin (1); A10, Carbenicillin (2); A11, Carbenicillin (3); A12, Carbenicillin (4); B1, Oxacillin (1); B2, Oxacillin (2); B3, Oxacillin (3); B4, Oxacillin (4); B5, Penimepicycline (1); B6, Penimepicycline (2); B7, Penimepicycline (3); B8, Penimepicycline (4); B9, Polymyxin B (1); B10, Polymyxin B (2); B11, Polymyxin B (3); B12, Polymyxin B (4); C1, Paromomycin (1); C2, Paromomycin (2); C3, Paromomycin (3); C4, Paromomycin (4); C5, Vancomycin (1); C6, Vancomycin (2); C7, Vancomycin (3); C8, Vancomycin (4); C9, D,L-Serinehydroxamate (1); C10, D,L-Serine hydroxamate (2); C11, D,L-Serine hydroxamate (3); C12, D,L-Serine hydroxamate (4); D1, Sisomicin (1); D2, Sisomicin (2); D3, Sisomicin (3); D4, Sisomicin (4); D5, Sulfamethazine (1); D6, Sulfamethazine (2); D7, Sulfamethazine (3); D8, Sulfamethazine (4); D9, Novobiocin (1); D10, Novobiocin (2); D11, Novobiocin (3); D12, Novobiocin (4); E1, 2,4-Diamino-6,7-diisopropylpteridine (1); E2, 2,4-Diamino-6,7-diisopropylpteridine (2); E3, 2,4-Diamino-6,7-diisopropylpteridine (3); E4, 2,4-Diamino-6,7-diisopropylpteridine (4); E5, Sulfadiazine (1); E6, Sulfadiazine (2); E7, Sulfadiazine (3); E8, Sulfadiazine (4); E9, Benzethoniumchloride (1); E10, Benzethoniumchloride (2); E11, Benzethoniumchloride (3); E12, Benzethoniumchloride (4); F1, Tobramycin (1); F2, Tobramycin (2); F3, Tobramycin (3); F4, Tobramycin (4); F5, Sulfathiazole (1); F6, Sulfathiazole (2); F7, Sulfathiazole (3); F8, Sulfathiazole (4); F9, 5-Fluoroorotic acid (1); F10, 5-Fluoroorotic acid (2); F11, 5-Fluoroorotic acid (3); F12, 5-Fluoroorotic acid (4); G1, Spectinomycin (1); G2, Spectinomycin (2); G3, Spectinomycin (3); G4, Spectinomycin (4); G5, Sulfamethoxazole (1); G6, Sulfamethoxazole (2); G7, Sulfamethoxazole (3); G8, Sulfamethoxazole (4); G9, L-Aspartic-β-hydroxamate (1); G10, L-Aspartic-β-hydroxamate (2); G11, L-Aspartic-β-hydroxamate (3); G12, L-Aspartic-β-hydroxamate (4); H1, Spiramycin (1); H2, Spiramycin (2); H3, Spiramycin (3); H4, Spiramycin (4); H5, Rifampicin (1); H6, Rifampicin (2); H7, Rifampicin (3); H8, Rifampicin (4); H9, Dodecyltrimethyl ammonium bromide (1); H10, Dodecyltrimethyl ammonium bromide (2); H11, Dodecyltrimethyl ammonium bromide (3); H12. Dodecyltrimethyl ammonium bromide (4). |
| PM13B – chemical | A1, Ampicillin (1); A2, Ampicillin (2); A3, Ampicillin (3); A4, Ampicillin (4); A5, Dequalinium chloride (1); A6, Dequalinium chloride (2); A7, Dequalinium chloride (3); A8, Dequalinium chloride (4); A9, Nickel chloride (1); A10, Nickel chloride (2); A11, Nickel chloride (3); A12, Nickel chloride (4); B1, Azlocillin (1); B2, Azlocillin (2); B3, Azlocillin (3); B4, Azlocillin (4); B5, 2, 2'-Dipyridyl (1); B6, 2, 2'-Dipyridyl (2); B7, 2, 2'-Dipyridyl (3); B8, 2, 2'-Dipyridyl (4); B9, Oxolinic acid (1); B10, Oxolinic acid (2); B11, Oxolinic acid (3); B12, Oxolinic acid (4); C1, 6-Mercaptopurine (1); C2, 6-Mercaptopurine (2); C3, -Mercaptopurine (3); C4, 6-Mercaptopurine (4); C5, Doxycycline (1); C6, Doxycycline (2); C7, Doxycycline (3); C8, Doxycycline (4); C9, Potassium chromate (1); C10,  Potassium chromate (2); C11, Potassium chromate (3); C12, Potassium chromate (4); D1, Cefuroxime (1); D2, Cefuroxime (2); D3, Cefuroxime (3); D4, Cefuroxime (4); D5, 5-Fluorouracil (1); D6, 5-Fluorouracil (2); D7, 5-Fluorouracil (3); D8, 5-Fluorouracil (4); D9, Rolitetracycline (1); D10, Rolitetracycline (2); D11, Rolitetracycline (3); D12, Rolitetracycline (4); E1, Cytosine-1- βD-arabinofuranoside (1); E2, Cytosine-1- βD-arabinofuranoside (2); E3, Cytosine-1-βD-arabinofuranoside (3); E4, Cytosine-1-βD-arabinofuranoside (4); E5, Geneticin (G418) (1); E6, Geneticin (G418) (2); E7, Geneticin (G418) (3); E8, Geneticin (G418) (4); E9, Ruthenium red (1); E10, Ruthenium red (2); E11, Ruthenium red (3); E12, Ruthenium red (4); F1, Cesium chloride (1); F2, Cesium chloride (2); F3, Cesium chloride (3); F4, Cesium chloride (4); F5, Glycine (1); F6, Glycine (2); F7, Glycine (3); F8, Glycine (4); F9, Thallium (I) acetate (1); F10, Thallium (I) acetate (2); F11, Thallium (I) acetate (3); F12, Thallium (I) acetate (4); G1, Cobalt chloride (1); G2, Cobalt chloride (2); G3, Cobalt chloride (3); G4, Cobalt chloride (4); G5, Manganese chloride (1); G6, Manganese chloride (2); G7, Manganese chloride (3); G8, Manganese chloride (4); G9, Trifluoperazine (1); G10 ,Trifluoperazine (2); G11, Trifluoperazine (3); G12, Trifluoperazine (4); H1, Cupric chloride (1); H2, Cupric chloride (2); H3, Cupric chloride (3); H4, Cupric chloride (4); H5, Moxalactam (1); H6, Moxalactam (2); H7, Moxalactam (3); H8, Moxalactam (4); H9, Tylosin (1); H10, Tylosin (2); H11, Tylosin (3); H12, Tylosin (4). |
| PM14A – chemical | A1, Acriflavine (1); A2, Acriflavine (2); A3, Acriflavine (3); A4, Acriflavine (4); A5, Furaltadone (1); A6, Furaltadone (2); A7, Furaltadone (3); A8, Furaltadone (4); A9, Sanguinarine (1); A10, Sanguinarine (2); A11, Sanguinarine (3); A12, Sanguinarine (4); B1, 9-Aminoacridine (1); B2, 9-Aminoacridine (2); B3, 9-Aminoacridine (3); B4, 9-Aminoacridine (4); B5, Fusaric acid (1); B6, Fusaric acid (2); B7, Fusaric acid (3); B8, Fusaric acid (4); B9, Sodium arsenate (1); B10, Sodium arsenate (2); B11, Sodium arsenate (3); B12, Sodium arsenate (4); C1, Boric Acid (1); C2, Boric Acid (2); C3, Boric Acid (3); C4, Boric Acid (4); C5, 1-Hydroxypyridine-2-thione (1); C6, 1-Hydroxypyridine-2-thione (2); C7, 1-Hydroxypyridine-2-thione (3); C8, 1-Hydroxypyridine-2-thione (4); C9, Sodium cyanate (1); C10, Sodium cyanate (2); C11, Sodium cyanate (3); C12, Sodium cyanate (4); D1, Cadmium chloride (1); D2, Cadmium chloride (2); D3, Cadmium chloride (3); D4, Cadmium chloride (4); D5, Iodoacetate (1); D6, Iodoacetate (2); D7, Iodoacetate (3); D8, Iodoacetate (4); D9, Sodium dichromate (1); D10, Sodium dichromate (2); D11, Sodium dichromate (3); D12, Sodium dichromate (4); E1, Cefoxitin (1); E2, Cefoxitin (2); E3, Cefoxitin (3); E4, Cefoxitin (4); E5, Nitrofurantoin (1); E6, Nitrofurantoin (2); E7, Nitrofurantoin (3); E8, Nitrofurantoin (4); E9, Sodium metaborate (1); E10, Sodium metaborate (2); E11, Sodium metaborate (3); E12, Sodium metaborate (4); F1, Chloramphenicol (1); F2, Chloramphenicol (2); F3, Chloramphenicol (3); F4, Chloramphenicol (4); F5, Piperacillin (1); F6, Piperacillin (2); F7, Piperacillin (3); F8, Piperacillin (4); F9, Sodium metavanadate (1); F10, Sodium metavanadate (2); F11, Sodium metavanadate (3); F12, Sodium metavanadate (4); G1, Chelerythrine (1); G2, Chelerythrine (2); G3, Chelerythrine (3); G4, Chelerythrine (4); G5, Carbenicillin (1); G6, Carbenicillin (2); G7, Carbenicillin (3); G8, Carbenicillin (4); G9, Sodium nitrite (1); G10, Sodium nitrite (2); G11, Sodium nitrite (3); G12, Sodium nitrite (4); H1 ,EGTA (1); H2, EGTA (2); H3, EGTA (3); H4, EGTA (4); H5, Promethazine (1); H6, Promethazine (2); H7, Promethazine (3); H8, Promethazine (4); H9, Sodium orthovanadate (1); H10, Sodium orthovanadate (2); H11, Sodium orthovanadate (3); H12, Sodium orthovanadate (4). |
| PM15B – chemical | A1, Procaine (1) ;A2, Procaine (2); A3, Procaine (3); A4, Procaine (4); A5, Guanidine hydrochloride (1); A6, Guanidine hydrochloride (2); A7, Guanidine hydrochloride (3); A8, Guanidine hydrochloride (4); A9, Cefmetazole (1); A10, Cefmetazole (2); A11, Cefmetazole (3); A12, Cefmetazole (4); B1, D-Cycloserine (1); B2, D-Cycloserine (2); B3, D-Cycloserine (3); B4, D-Cycloserine (4); B5, EDTA (1); B6, EDTA (2); B7, EDTA (3); B8, EDTA (4); B9, 5,7-Dichloro- 8-hydroxyquinaldine (1); B10, 5,7-Dichloro- 8-hydroxyquinaldine (2); B11, 5,7-Dichloro- 8-hydroxyquinaldine (3); B12, 5,7-Dichloro- 8-hydroxyquinaldine (4); C1, 5,7-Dichloro-8-hydroxyquinoline (1); C2, 5,7-Dichloro-8-hydroxyquinoline (2); C3, 5,7-Dichloro-8-hydroxyquinoline (3); C4, 5,7-Dichloro-8-hydroxyquinoline (4); C5, Fusidic acid (1); C6, Fusidic acid (2); C7, Fusidic acid (3); C8, Fusidic acid (4); C9, 1,10-Phenanthroline (1); C10, 1,10-Phenanthroline (2); C11, 1,10-Phenanthroline (3); C12, 1,10-Phenanthroline (4); D1, Phleomycin (1); D2, Phleomycin (2); D3, Phleomycin (3); D4, Phleomycin (4); D5, Domiphen bromide (1); D6, Domiphen bromide (2); D7, Domiphen bromide (3); D8, Domiphen bromide (4); D9, Nordihydroguaia retic acid (1); D10, Nordihydroguaia retic acid (2); D11, Nordihydroguaia retic acid (3); D12, Nordihydroguaia retic acid (4); E1, Alexidine (1); E2, Alexidine (2); E3, Alexidine (3); E4, Alexidine (4); E5, 5-Nitro-2-furaldehyde semicarbazone (1); E6, 5-Nitro-2-furaldehyde semicarbazone (2); E7, 5-Nitro-2-furaldehyde semicarbazone (3); E8, 5-Nitro-2-furaldehyde semicarbazone (4); E9, Methyl viologen (1); E10, Methyl viologen (2); E11, Methyl viologen (3); E12, Methyl viologen (4); F1, 3, 4-Dimethoxybenzyl alcohol (1); F2, 3, 4-Dimethoxybenzyl alcohol (2); F3, 3, 4-Dimethoxybenzyl alcohol (3); F4, 3, 4-Dimethoxybenzyl alcohol (4); F5, Oleandomycin (1); F6, Oleandomycin (2); F7, Oleandomycin (3); F8, Oleandomycin (4); F9, Puromycin (1); F10, Puromycin (2); F11, Puromycin (3); F12, Puromycin (4); G1, CCCP (1); G2, CCCP (2); G3, CCCP (3); G4, CCCP (4); G5, Sodium azide (1); G6, Sodium azide (2); G7, Sodium azide (3); G8, Sodium azide (4); G9, Menadione (1); G10, Menadione (2); G11, Menadione (3); G12, Menadione (4); H1, 2-Nitroimidazole (1); H2, 2-Nitroimidazole (2); H3, 2-Nitroimidazole (3); H4, 2-Nitroimidazole (4); H5, Hydroxyurea (1); H6, Hydroxyurea (2); H7, Hydroxyurea (3); H8, Hydroxyurea (4); H9, Zinc chloride (1); H10, Zinc chloride (2); H11, Zinc chloride (3); H12, Zinc chloride (4). |
| PM16A – chemical | A1, Cefotaxime (1); A2, Cefotaxime (2); A3, Cefotaxime (3); A4, Cefotaxime (4); A5, Phosphomycin (1); A6, Phosphomycin (2); A7, Phosphomycin (3); A8, Phosphomycin (4); A9, 5-Chloro-7-iodo-8-hydroxyquinoline (1); A10, 5-Chloro-7-iodo-8-hydroxyquinoline (2); A11, 5-Chloro-7-iodo-8-hydroxyquinoline (3); A12, 5-Chloro-7-iodo-8-hydroxyquinoline (4); B1, Norfloxacin (1); B2, Norfloxacin (2); B3, Norfloxacin (3); B4, Norfloxacin (4); B5, Sulfanilamide (1); B6, Sulfanilamide (2); B7, Sulfanilamide (3); B8, Sulfanilamide (4); B9, Trimethoprim (1); B10, Trimethoprim (2); B11, Trimethoprim (3); B12, Trimethoprim (4); C1, Dichlofluanid (1); C2, Dichlofluanid (2); C3, Dichlofluanid (3); C4, Dichlofluanid (4); C5, Protamine sulfate (1); C6, Protamine sulfate (2); C7, Protamine sulfate (3); C8, Protamine sulfate (4); C9, Cetylpyridinium chloride (1); C10, Cetylpyridinium chloride (2); C11, Cetylpyridinium chloride (3); C12, Cetylpyridinium chloride (4); D1, 1-Chloro -2,4-dinitrobenzene (1); D2, 1-Chloro -2,4-dinitrobenzene (2); D3, 1-Chloro -2,4-dinitrobenzene (3); D4, 1-Chloro -2,4-dinitrobenzene (4); D5, Diamide (1); D6, Diamide (2); D7, Diamide (3); D8, Diamide (4); D9, Cinoxacin (1); D10, Cinoxacin (2); D11, Cinoxacin (3); D12, Cinoxacin (4); E1, Streptomycin (1); E2, Streptomycin (2); E3, Streptomycin (3); E4, Streptomycin (4); E5, 5-Azacytidine (1); E6, 5-Azacytidine (2); E7, 5-Azacytidine (3); E8, 5-Azacytidine (4); E9, Rifamycin SV (1); E10, Rifamycin SV (2); E11, Rifamycin SV (3); E12, Rifamycin SV (4); F1, Potassium tellurite (1); F2, Potassium tellurite (2); F3, Potassium tellurite (3); F4, Potassium tellurite (4); F5, Sodium selenite (1); F6, Sodium selenite (2); F7, Sodium selenite (3); F8, Sodium selenite (4); F9, Aluminum sulfate (1); F10, Aluminum sulfate (2); F11, Aluminum sulfate (3); F12, Aluminum sulfate (4); G1, Chromium chloride (1); G2, Chromium chloride (2); G3, Chromium chloride (3); G4, Chromium chloride (4); G5, Ferric chloride (1); G6, Ferric chloride (2); G7, Ferric chloride (3); G8, Ferric chloride (4); G9, L-Glutamic-ghydroxamate (1); G10, L-Glutamic-ghydroxamate (2); G11, L-Glutamic-ghydroxamate (3); G12, L-Glutamic-ghydroxamate (4); H1, Glycine hydroxamate (1); H2, Glycine hydroxamate (2); H3, Glycine hydroxamate (3); H4, Glycine hydroxamate (4); H5, Chloroxylenol (1); H6, Chloroxylenol (2); H7, Chloroxylenol (3); H8, Chloroxylenol (4); H9, Sorbic acid (1); H10, Sorbic acid (2); H11, Sorbic acid (3); H12, Sorbic acid (4). |
| PM17A – chemical | A1, D-Serine (1); A2, D-Serine (2); A3, D-Serine (3); A4, D-Serine (4); A5, β-ChloroL-alanine hydrochloride (1); A6, β-ChloroL-alanine hydrochloride (2); A7, β-ChloroL-alanine hydrochloride (3); A8, β-ChloroL-alanine hydrochloride (4); A9, Thiosalicylic acid (1); A10, Thiosalicylic acid (2); A11, Thiosalicylic acid (3); A12, Thiosalicylic acid (4); B1, Sodium salicylate (1); B2, Sodium salicylate (2); B3, Sodium salicylate (3); B4, Sodium salicylate (4); B5, Hygromycin B (1); B6, Hygromycin B (2); B7, Hygromycin B (3); B8, Hygromycin B (4); B9, Ethionamide (1); B10, Ethionamide (2); B11, Ethionamide (3); B12, Ethionamide (4); C1, 4-Aminopyridine (1); C2, 4-Aminopyridine (2); C3, 4-Aminopyridine (3); C4, 4-Aminopyridine (4); C5, Sulfachloropyridazine (1); C6, Sulfachloropyridazine (2); C7, Sulfachloropyridazine (3); C8, Sulfachloropyridazine (4); C9, Sulfamonomethoxine (1); C10, Sulfamonomethoxine (2); C11, Sulfamonomethoxine (3); C12, Sulfamonomethoxine (4); D1, Oxycarboxin (1); D2, Oxycarboxin (2); D3, Oxycarboxin (3); D4, Oxycarboxin (4); D5, 3-Amino-1,2,4-triazole (1); D6, 3-Amino-1,2,4-triazole (2); D7, 3-Amino-1,2,4-triazole (3); D8, 3-Amino-1,2,4-triazole (4); D9, Chlorpromazine (1); D10, Chlorpromazine (2); D11, Chlorpromazine (3); D12, Chlorpromazine (4); E1, Niaproof (1); E2, Niaproof (2); E3, Niaproof (3); E4, Niaproof (4); E5, Compound 48/80 (1); E6, Compound 48/80 (2); E7, Compound 48/80 (3); E8, Compound 48/80 (4); E9, Sodium tungstate (1); E10, Sodium tungstate (2); E11, Sodium tungstate (3); E12, Sodium tungstate (4); F1, Lithium chloride (1); F2, Lithium chloride (2)  F3, Lithium chloride (3); F4, Lithium chloride (4); F5, DL-Methionine hydroxamate (1); F6, DL-Methionine hydroxamate (2); F7, DL-Methionine hydroxamate (3); F8, DL-Methionine hydroxamate (4); F9, Tannic acid (1); F10, Tannic acid (2); F11, Tannic acid (3); F12, Tannic acid (4); G1, Chlorambucil (1); G2, Chlorambucil (2); G3, Chlorambucil (3); G4, Chlorambucil (4); G5, Cefamandole nafate (1); G6, Cefamandole nafate (2); G7, Cefamandole nafate (3); G8, Cefamandole nafate (4); G9, Cefoperazone (1); G10, Cefoperazone (2); G11, Cefoperazone (3); G12, Cefoperazone (4); H1, Cefsulodin (1); H2, Cefsulodin (2); H3, Cefsulodin (3); H4, Cefsulodin (4); H5, Caffeine (1); H6, Caffeine (2); H7, Caffeine (3); H8, Caffeine (4); H9, Phenylarsine oxide (1); H10, Phenylarsine oxide (2); H11, Phenylarsine oxide (3); H12, Phenylarsine oxide (4). |
| PM18C – chemical | A1, Ketoprofen (1); A2, Ketoprofen (2); A3, Ketoprofen (3); A4, Ketoprofen (4); A5, Sodium pyrophosphate decahydrate (1); A6, Sodium pyrophosphate decahydrate (2); A7, Sodium pyrophosphate decahydrate (3); A8, Sodium pyrophosphate decahydrate (4); A9, Thiamphenicol (1); A10, Thiamphenicol (2); A11, Thiamphenicol (3); A12, Thiamphenicol (4); B1, Trifluorothymidine (1); B2, Trifluorothymidine (2); B3, Trifluorothymidine (3); B4, Trifluorothymidine (4); B5, Pipemidic Acid (1); B6, Pipemidic Acid (2); B7, Pipemidic Acid (3); B8, Pipemidic Acid (4); B9, Azathioprine (1); B10, Azathioprine (2); B11, Azathioprine (3); B12, Azathioprine (4); C1, Poly-L-lysine (1); C2, Poly-L-lysine (2); C3, Poly-L-lysine (3); C4, Poly-L-lysine (4); C5, Sulfisoxazole (1); C6, Sulfisoxazole (2); C7, Sulfisoxazole (3); C8, Sulfisoxazole (4); C9, Pentachlorophenol (1); C10, Pentachlorophenol (2); C11, Pentachlorophenol (3); C12, Pentachlorophenol (4); D1, Sodium m-arsenite (1); D2, Sodium m-arsenite (2); D3, Sodium m-arsenite (3); D4, Sodium m-arsenite (4); D5, Sodium bromate (1); D6, Sodium bromate (2); D7, Sodium bromate (3); D8, Sodium bromate (4); D9, Lidocaine (1); D10, Lidocaine (2); D11, Lidocaine (3); D12, Lidocaine (4); E1, Sodium metasilicate (1); E2, Sodium metasilicate (2); E3, Sodium metasilicate (3); E4, Sodium metasilicate (4); E5, Sodium m-periodate (1); E6, Sodium m-periodate (2); E7, Sodium m-periodate (3); E8, Sodium m-periodate (4); E9, Antimony (III) chloride (1); E10, Antimony (III) chloride (2); E11, Antimony (III) chloride (3); E12, Antimony (III) chloride (4); F1, Semicarbazide (1); F2, Semicarbazide (2); F3, Semicarbazide (3); F4, Semicarbazide (4); F5, Tinidazole (1); F6, Tinidazole (2); F7, Tinidazole (3); F8, Tinidazole (4); F9, Aztreonam (1); F10, Aztreonam (2); F11, Aztreonam (3); F12, Aztreonam (4); G1, Triclosan (1); G2, Triclosan (2); G3, Triclosan (3); G4, Triclosan (4); G5, 3,5-Diamino-1,2,4-triazole (Guanazole) (1); G6, 3,5-Diamino-1,2,4-triazole (Guanazole) (2); G7, 3,5-Diamino-1,2,4-triazole (Guanazole) (3); G8, 3,5-Diamino-1,2,4-triazole (Guanazole) (4); G9, Myricetin (1); G10, Myricetin (2); G11, Myricetin (3); G12, Myricetin (4); H1, 5-fluoro-5’-deoxyuridine (1); H2, 5-fluoro-5’-deoxyuridine (2); H3, 5-fluoro-5’-deoxyuridine (3); H4, 5-fluoro-5’-deoxyuridine (4); H5, 2-Phenylphenol (1); H6, 2-Phenylphenol (2); H7, 2-Phenylphenol (3); H8, 2-Phenylphenol (4); H9, Plumbagin (1); H10, Plumbagin (2); H11, Plumbagin (3); H12, Plumbagin (4). |
| PM19 – chemical | A1, Josamycin (1); A2, Josamycin (2); A3, Josamycin (3); A4, Josamycin (4); A5, Gallic acid (1); A6, Gallic acid (2); A7, Gallic acid (3); A8, Gallic acid (4); A9, Coumarin (1); A10, Coumarin (2); A11, Coumarin (3); A12, Coumarin (4); B1, Methyltrioctylammonium chloride (1); B2, Methyltrioctylammonium chloride (2); B3, Methyltrioctylammonium chloride (3); B4, Methyltrioctylammonium chloride (4); B5, Harmane (1); B6, Harmane (2); B7, Harmane (3); B8, Harmane (4); B9, 2,4-Dintrophenol (1); B10, 2,4-Dintrophenol (2); B11, 2,4-Dintrophenol (3); B12, 2,4-Dintrophenol (4); C1, Chlorhexidine (1); C2, Chlorhexidine (2); C3, Chlorhexidine (3); C4, Chlorhexidine (4); C5, Umbelliferone (1); C6, Umbelliferone (2); C7, Umbelliferone (3); C8, Umbelliferone (4); C9, Cinnamic acid (1); C10, Cinnamic acid (2); C11, Cinnamic acid (3); C12, Cinnamic acid (4); D1, Disulphiram (1); D2, Disulphiram (2); D3, Disulphiram (3); D4, Disulphiram (4); D5, Iodonitro Tetrazolium Violet (1); D6, Iodonitro Tetrazolium Violet (2); D7, Iodonitro Tetrazolium Violet (3); D8, Iodonitro Tetrazolium Violet (4); D9, Phenyl- methylsulfonylfluoride (PMSF) (1); D10, Phenyl- methylsulfonylfluoride (PMSF) (2); D11, Phenyl- methylsulfonylfluoride (PMSF) (3); D12, Phenyl- methylsulfonylfluoride (PMSF) (4); E1, FCCP (1); E2, FCCP (2); E3, FCCP (3); E4, FCCP (4); E5, D,L-Thioctic Acid (1); E6, D,L-Thioctic Acid (2); E7, D,L-Thioctic Acid (3); E8, D,L-Thioctic Acid (4); E9, Lawsone (1); E10, Lawsone (2); E11, Lawsone (3); E12, Lawsone (4); F1, Phenethicillin (1); F2, Phenethicillin (2); F3, Phenethicillin (3); F4, Phenethicillin (4); F5, Blasticidin S (1); F6, Blasticidin S (2); F7, Blasticidin S (3); F8, Blasticidin S (4); F9, Sodium caprylate (1); F10, Sodium caprylate (2); F11, Sodium caprylate (3); F12, Sodium caprylate (4); G1, Lauryl sulfobetaine (1); G2, Lauryl sulfobetaine (2); G3, Lauryl sulfobetaine (3); G4, Lauryl sulfobetaine (4); G5, Dihydrostreptomycin (1); G6, Dihydrostreptomycin (2); G7, Dihydrostreptomycin (3); G8, Dihydrostreptomycin (4); G9, Hydroxylamine (1); G10, Hydroxylamine (2); G11, Hydroxylamine (3); G12, Hydroxylamine (4); H1, Hexammine cobalt (III) chloride (1); H2, Hexammine cobalt (III) chloride (2); H3, Hexammine cobalt (III) chloride (3); H4, Hexammine cobalt (III) chloride (4); H5, Thioglycerol (1); H6, Thioglycerol (2); H7, Thioglycerol (3); H8, Thioglycerol (4); H9, Polymyxin B (1); H10, Polymyxin B (2); H11, Polymyxin B (3); H12, Polymyxin B (4). |
| PM20B – chemical | A1, Amitriptyline (1); A2, Amitriptyline (2); A3, Amitriptyline (3) A4, Amitriptyline (4); A5, Apramycin (1); A6, Apramycin(2); A7, Apramycin (3); A8, Apramycin (4); A9, Benserazide (1); A10, Benserazide (2); A11, Benserazide (3); A12, Benserazide (4); B1, Orphenadrine (1); B2, Orphenadrine (2); B3, Orphenadrine (3); B4, Orphenadrine (4); B5, D,L-Propranolol (1); B6, D,L-Propranolol (2); B7, D,L-Propranolol (3), B8, D,L-Propranolol (4); B9, Tetrazolium Violet (1); B10, Tetrazolium Violet (2); B11, Tetrazolium Violet (3); B12, Tetrazolium Violet (4); C1, Thioridazine (1); C2, Thioridazine (2); C3, Thioridazine (3); C4, Thioridazine (4); C5, Atropine (1); C6, Atropine (2); C7, Atropine (3); C8, Atropine (4); C9, Ornidazole (1); C10, Ornidazole (2); C11, Ornidazole (3); C12, Ornidazole (4); D1, Proflavine (1); D2, Proflavine (2); D3, Proflavine (3); D4, Proflavine (4); D5, Ciprofloxacin (1); D6, Ciprofloxacin (2); D7, Ciprofloxacin (3); D8, Ciprofloxacin (4); D9, 18-Crown-6 Ether (1); D10, 18-Crown-6 Ether (2); D11, 18-Crown-6 Ether (3); D12, 18-Crown-6 Ether (4); E1, Crystal violet (1); E2, Crystal violet (2); E3, Crystal violet (3); E4, Crystal violet (4); E5, Dodine (1); E6, Dodine (2); E7, Dodine (3); E8, Dodine (4); E9, Hexachlorophene (1); E10, Hexachlorophene (2); E11, Hexachlorophene (3); E12, Hexachlorophene (4); F1, 4-Hydroxycoumarin (1); F2, 4-Hydroxycoumarin (2); F3, 4-Hydroxycoumarin (3); F4, 4-Hydroxycoumarin (4); F5, Oxytetracycline (1); F6, Oxytetracycline (2); F7, Oxytetracycline (3); F8, Oxytetracycline (4); F9, Pridinol (1); F10, Pridinol (2); F11, Pridinol (3); F12, Pridinol (4); G1, Captan (1); G2, Captan (2); G3, Captan (3); G4, Captan (4); G5, 3,5-Dinitrobenzene (1); G6, 3,5-Dinitrobenzene (2); G7, 3,5-Dinitrobenzene (3); G8, 3,5-Dinitrobenzene (4); G9, 8-Hydroxyquinoline (1); G10, 8-Hydroxyquinoline (2); G11, 8-Hydroxyquinoline (3); G12, 8-Hydroxyquinoline (4); H1, Patulin (1); H2, Patulin (2); H3, Patulin (3); H4, Patulin (4); H5, Tolylfluanid (1); H6, Tolylfluanid (2); H7, Tolylfluanid (3); H8, Tolylfluanid (4); H9, Troleandomycin (1); H10, Troleandomycin (2); H11, Troleandomycin (3); H12, Troleandomycin (4). |

**Supplementary Table 2. Genes missing from pCROD1 of *C. rodentium* ICC180**

| **Gene** | **Location** | **Function** |
| --- | --- | --- |
| *ROD_RS25055* | 240..494 | Replication regulatory protein repA2 |
| *ROD_RS25060* | 797..1654 | Replication protein |
| *ROD_RS25065* | 2593..3246 | Hypothetical protein |
| *ROD_RS25070* | 3339..3596 | Antitoxin |
| *ROD_RS25075* | 3598..3930 | Hypothetical protein |
| *ROD_RS25080* | 4318..4614 | Transposase |
| *ROD_RS25085* | 5726..6955 | Autotransporter strand-loop-strand |
| *ROD_RS25090* | 6939..11720 | Autotransporter |
| *ROD_RS25095* | 12358..12558 | Hypothetical protein |
| *ROD_RS25100* | 12814..13043 | Transposase |
| *ROD_RS25105* | 14045..14563 | Fimbrial protein |
| *ROD_RS25110* | 14636..17053 | Fimbrial protein |
| *ROD_RS25115* | 17046..17738 | Fimbrial protein |
| *ROD_RS25120* | 18251..18820 | Hypothetical protein |
| *ROD_RS25125* | 18945..19904 | Hypothetical protein |
| *ROD_RS25130* | 20068..20844 | EAL domain-containing protein |
| *ROD_RS25135* | 22328..26254 | Autotransporter |
| *ROD_RS25140* | 26743..26993 | Toxin HigB-2 |
| *ROD_RS25145* | 27079..27339 | Transcriptional regulator |
| *ROD_RS25150* | 27957..30536 | Usher protein |
| *ROD_RS25155* | 30578..31048 | Hypothetical protein |
| *ROD_RS25160* | 33519..33941 | Twitching motility protein PilT |
| *ROD_RS25165* | 33938..34168 | Virulence factor |
| *ROD_RS25170* | 34837..35055 | Hypothetical protein |
| *ROD_RS25175* | 35057..35362 | Hypothetical protein |
| *ROD_RS25180* | 35364..35690 | Hypothetical protein |
| *ROD_RS25185* | 35680..36471 | Resolvase |
| *ROD_RS25190* | 36627..40730 | Autotransporter |
| *ROD_RS25730* | 41808..43358 | Hypothetical protein |
| *ROD_RS25205* | 43907..44329 | Entry exclusion protein 2 |
| *ROD_RS25210* | 44567..45523 | Hypothetical protein |
| *ROD_RS25215* | 45875..46504 | Serine recombinase |
| *ROD_RS25220* | 46779..47306 | Putative resolvase |
| *ROD_RS25225* | 47600..48241 | Chromosome partitioning protein ParA |
| *ROD_RS25230* | 48333..48665 | Molecular chaperone GroEL |
| *ROD_RS25235* | 49280..50002 | DNA repair protein |
| *ROD_RS25240* | 50082..51653 | Transposase |
| *ROD_RS25250* | 52020..52697 | Transposase |
| *ROD_RS25255* | 52721..52750 | Endonuclease |
| *ROD_RS25260* | 53458..54144 | Hypothetical protein |
| *ROD_RS25265* | 54141..54449 | Hypothetical protein |
