# Supplementary material for: The in vitro and in vivo effects of constitutive light expression on a bioluminescent strain of the mouse enteropathogen Citrobacter rodentium
Source: PeerJ. 2016 Jun 22;4:e2130. doi: 10.7717/peerj.2130 (PMC4924136; doi:10.7717/peerj.2130)
Supplement: Table S2 — List of genes missing from plasmid pCROD1 of C. rodentium ICC180, as determined by sequencing. [file peerj-04-2130-s003.docx]

**Supplementary Table 2. Genes missing from pCROD1 of *C. rodentium* ICC180**

List of genes missing from plasmid pCROD1 of *C. rodentium* ICC180, as determined by sequencing

| **Gene** | **Location** | **Function** |
| --- | --- | --- |
| *ROD_RS25055* | 240..494 | Replication regulatory protein repA2 |
| *ROD_RS25060* | 797..1654 | Replication protein |
| *ROD_RS25065* | 2593..3246 | Hypothetical protein |
| *ROD_RS25070* | 3339..3596 | Antitoxin |
| *ROD_RS25075* | 3598..3930 | Hypothetical protein |
| *ROD_RS25080* | 4318..4614 | Transposase |
| *ROD_RS25085* | 5726..6955 | Autotransporter strand-loop-strand |
| *ROD_RS25090* | 6939..11720 | Autotransporter |
| *ROD_RS25095* | 12358..12558 | Hypothetical protein |
| *ROD_RS25100* | 12814..13043 | Transposase |
| *ROD_RS25105* | 14045..14563 | Fimbrial protein |
| *ROD_RS25110* | 14636..17053 | Fimbrial protein |
| *ROD_RS25115* | 17046..17738 | Fimbrial protein |
| *ROD_RS25120* | 18251..18820 | Hypothetical protein |
| *ROD_RS25125* | 18945..19904 | Hypothetical protein |
| *ROD_RS25130* | 20068..20844 | EAL domain-containing protein |
| *ROD_RS25135* | 22328..26254 | Autotransporter |
| *ROD_RS25140* | 26743..26993 | Toxin HigB-2 |
| *ROD_RS25145* | 27079..27339 | Transcriptional regulator |
| *ROD_RS25150* | 27957..30536 | Usher protein |
| *ROD_RS25155* | 30578..31048 | Hypothetical protein |
| *ROD_RS25160* | 33519..33941 | Twitching motility protein PilT |
| *ROD_RS25165* | 33938..34168 | Virulence factor |
| *ROD_RS25170* | 34837..35055 | Hypothetical protein |
| *ROD_RS25175* | 35057..35362 | Hypothetical protein |
| *ROD_RS25180* | 35364..35690 | Hypothetical protein |
| *ROD_RS25185* | 35680..36471 | Resolvase |
| *ROD_RS25190* | 36627..40730 | Autotransporter |
| *ROD_RS25730* | 41808..43358 | Hypothetical protein |
| *ROD_RS25205* | 43907..44329 | Entry exclusion protein 2 |
| *ROD_RS25210* | 44567..45523 | Hypothetical protein |
| *ROD_RS25215* | 45875..46504 | Serine recombinase |
| *ROD_RS25220* | 46779..47306 | Putative resolvase |
| *ROD_RS25225* | 47600..48241 | Chromosome partitioning protein ParA |
| *ROD_RS25230* | 48333..48665 | Molecular chaperone GroEL |
| *ROD_RS25235* | 49280..50002 | DNA repair protein |
| *ROD_RS25240* | 50082..51653 | Transposase |
| *ROD_RS25250* | 52020..52697 | Transposase |
| *ROD_RS25255* | 52721..52750 | Endonuclease |
| *ROD_RS25260* | 53458..54144 | Hypothetical protein |
| *ROD_RS25265* | 54141..54449 | Hypothetical protein |
