# Supplementary figures and images for: The in vitro and in vivo effects of constitutive light expression on a bioluminescent strain of the mouse enteropathogen Citrobacter rodentium
Source: PeerJ. 2016 Jun 22;4:e2130. doi: 10.7717/peerj.2130 (PMC4924136; doi:10.7717/peerj.2130)

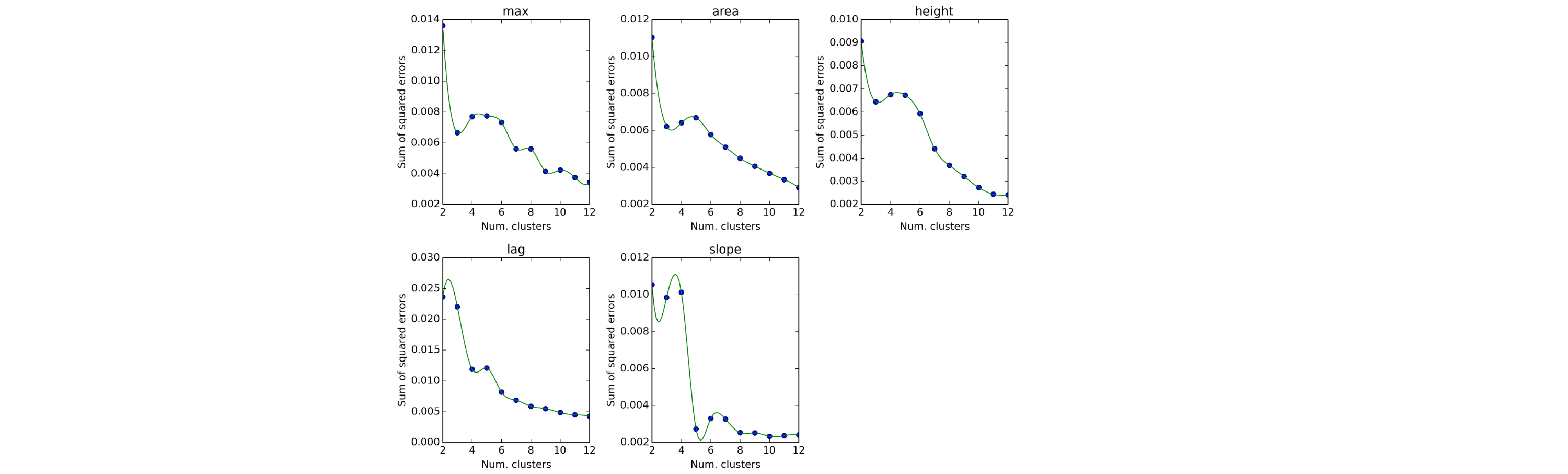

Supplement: Figure S1 — Data was analysed using the DuctApe software suite. [file peerj-04-2130-s004.png]

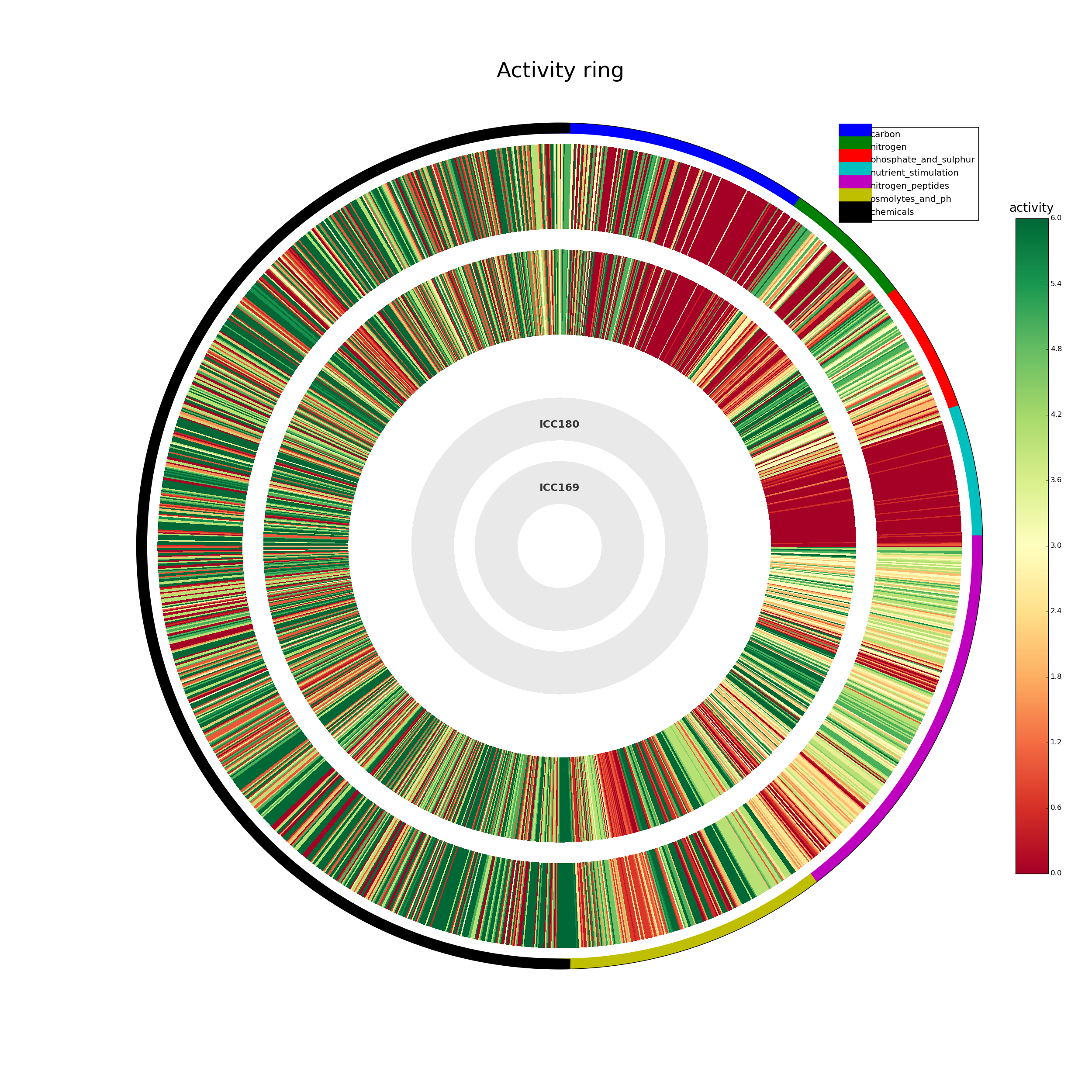

Supplement: Figure S2 — Wildtype C. rodentium ICC169 (shown as purple lines) and its bioluminescent derivative ICC180 (shown as blue lines) were grown on two separate occasions using PM plates 1–20 (categorised by colour [see Key]). Differences between the growth of ICC169 and ICC180 in each individual well were analysed using the moderated t-test provided by limma. Wells in which the differences had an adjusted p-value of less than 0.5 (stringent cut-off) are shown. [file peerj-04-2130-s005.png]

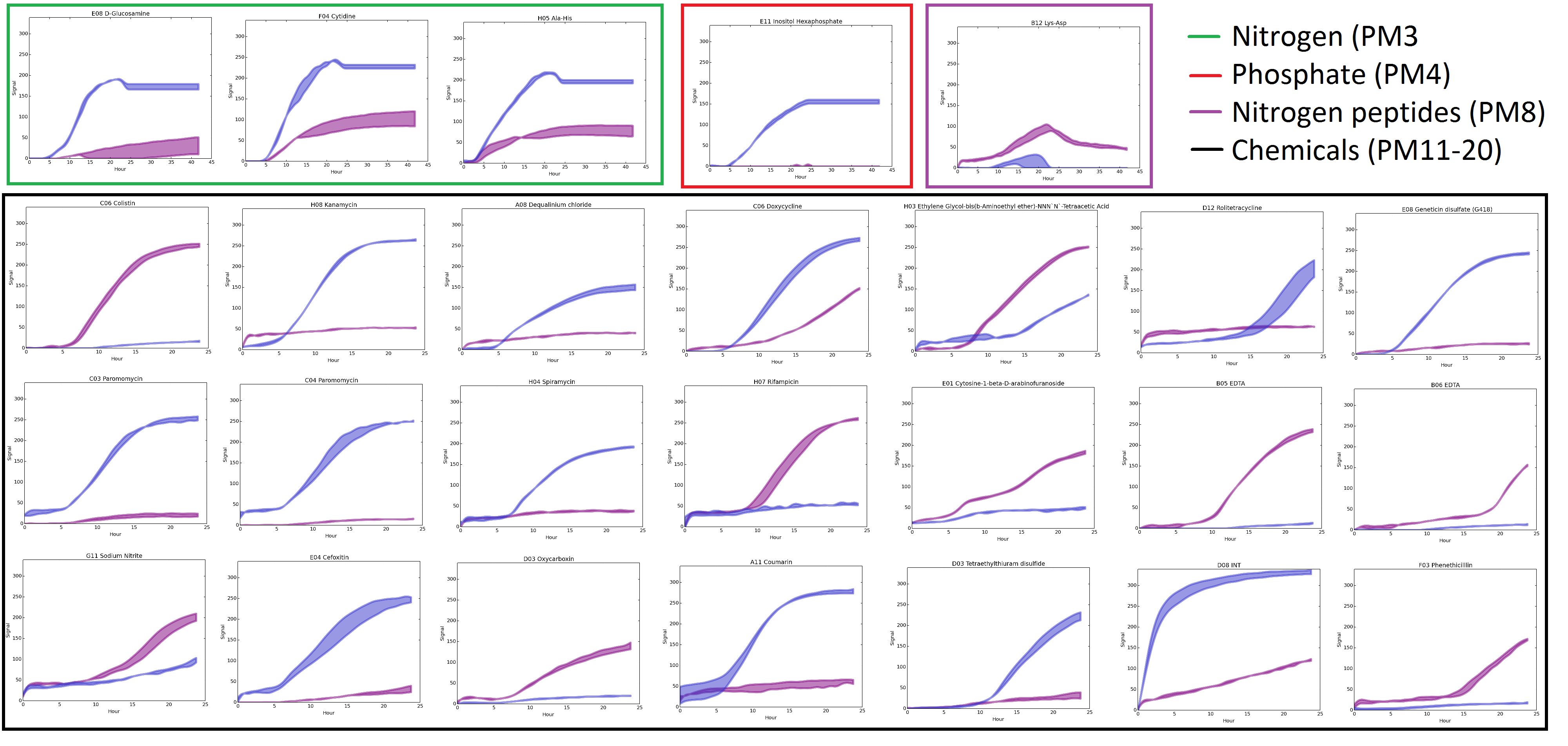

Supplement: Figure S3 — Groups of larvae (n = 10) of the Greater Wax Moth Galleria mellonella were infected with ∼108 CFU of C. rodentium ICC169 or ICC180 and monitored for bioluminescence using a plate luminometer. Data (medians with ranges) is presented from experiments performed on 3 separate occasions and is given as relative light units [RLU] waxworm−1. [file peerj-04-2130-s006.png]
